# Increased use of knowledge translation strategies is associated with greater research impact on public health policy and practice: an analysis of trials of nutrition, physical activity, sexual health, tobacco, alcohol and substance use interventions

**DOI:** 10.1186/s12961-022-00817-2

**Published:** 2022-01-31

**Authors:** Luke Wolfenden, Kaitlin Mooney, Sharleen Gonzalez, Alix Hall, Rebecca Hodder, Nicole Nathan, Serene Yoong, Elizabeth Ditton, Rachel Sutherland, Christophe Lecathelinais, Sam McCrabb

**Affiliations:** 1 School of Medicine and Public Health, Faculty of Health and Medicine, University of Newcastle, Newcastle, NSW 2318 Australia; 2 Hunter New England Population Health, Locked Bag 10, Hunter New England Local Health District, Wallsend, NSW 2287 Australia; 3 Hunter Medical Research Institute, Newcastle, NSW Australia

**Keywords:** Implementation science, Knowledge translation, Public health, Policy, Practice research impact

## Abstract

**Background:**

Greater use of knowledge translation (KT) strategies is recommended to improve the research impact of public health trials. The purpose of this study was to describe (1) the research impact of setting-based public health intervention trials on public health policy and practice; (2) the association between characteristics of trials and their research impact on public health policy and practice; and (3) the association between the use of KT strategies and research impacts on public health policy and practice.

**Methods:**

We conducted a survey of authors of intervention trials targeting nutrition, physical activity, sexual health, tobacco, alcohol or substance use. We assessed the use of KT strategies aligned to domains of the Knowledge-To-Action Framework. We defined “research impact” on health policy and practice as any one or more of the following: citation in policy documents or announcements, government reports, training materials, guidelines, textbooks or court rulings; or endorsement by a (non)governmental organization; use in policy or practice decision-making; or use in the development of a commercial resource or service.

**Results:**

Of the included trials, the authors reported that 65% had one or more research impacts. The most frequently reported research impact was citation in a policy document or announcement (46%). There were no significant associations between the effectiveness of the intervention, trial risk of bias, setting or health risk and trial impact. However, for every one unit increase in the total KT score (range 0–8), reflecting greater total KT activity, the odds of a health policy or practice research impact increased by approximately 30% (OR = 1.30, 95% CI: 1.02, 1.66; *p* = 0.031). Post hoc examination of KT domain scores suggests that KT actions focused on providing tailored support to facilitate program implementation and greater use of research products and tools to disseminate findings to end-users may be most influential in achieving impact.

**Conclusions:**

Trials of public health interventions frequently have public health impacts, and the use of more comprehensive KT strategies may facilitate greater research impact.

**Supplementary Information:**

The online version contains supplementary material available at 10.1186/s12961-022-00817-2.

## Background

Public health nutrition, physical activity, sexual health, tobacco, alcohol or substance use interventions can promote good health and prevent morbidity and mortality [1]. Public health research has a key role in improving community health and the prevention of disease. Indeed, the bedrock of the evidence-based medicine paradigm is the conduct and use of the best available scientific evidence to inform public health policy and practice to improve community health. Research impact frameworks [2] list a range of potential research impacts that health research could have on public health policy or practice, including government or industry adoption, commercialization or use to inform policy or court rulings.

Given the cost of public health interventions, the benefit of investment in public health research has been questioned [3, 4]. A study of 18 health promotion and primary prevention intervention trials funded by Australia’s National Health and Medical Research Council reported that just three (16%) had a research impact on public health policy and practice [5]. A case study analysis of 17 health promotion trials funded by a government demonstration grant scheme found that 10 (59%) had “moderate” to “high” policy or practice impacts, with the remaining having limited research impact [6]. While such estimates of research impact on public health policy and practice are highly variable, collectively they appear higher than commonly reported in other fields of medicine, which suggests that 14% of health innovations influence policy or practice, taking on average 17 years to do so [7–10]. This may be the result of differences between studies in methodological approaches to measuring research translation. Nonetheless, considerable scope remains to improve and better understand the impact of public health research.

Knowledge translation (KT) is a “*dynamic and iterative process that includes the synthesis, dissemination, exchange and ethically sound application of knowledge to improve health, provide more effective health services and products, and strengthen the health care system*” [11]. The actions of knowledge producers (e.g. researchers), and knowledge users (e.g. policy-makers) influence the likelihood that knowledge will be applied to improve health policy or practice. Improving KT is required to enhance the research impact of public health prevention intervention. A range of KT frameworks have been developed to support research translation, including the framework for knowledge translation [12] and the push–pull capacity model [13]. However, the most frequently cited and widely applied [14] is the Knowledge-to-Action (KTA) Framework [15]. The framework describes processes related to the creation of knowledge, such as generating evidence that is more applicable and user-friendly for end-users; and knowledge actions, such as activities to disseminate research findings to and promote the use of knowledge by end-users. Both knowledge producers and users have a role in successful KT, and their engagement across the KT processes is recommended.

A small number of studies have sought to describe the use of strategies to improve public health research translation. A national survey of United States public health researchers found that 34% always or usually involved stakeholders in the research process and 32% produced summaries of their research findings suitable for non-research audiences [16]. An international study of researchers in the United Kingdom, United States and Brazil found that two thirds of researchers reported using seminars or workshops, 48% face-to-face meetings, 39% media interviews and 12% targeted mailings to disseminate their research findings [17].

The likelihood of research translation may also vary according to characteristics of the research including its scientific quality, the setting, the health risk examined and the findings of the research [18–20]. For example, for intervention studies, the scientific quality of the research and evidence of the intervention having a beneficial impact are important considerations for public health policy-makers and practitioners when considering investments in public health interventions [18]. Furthermore, variation in research use is also evident across different public health disciplines, content areas and settings [19, 20]. Understanding the characteristics of research associated with impact may help to identify where research impact is more likely and where additional strategies may be required to support translation of evidence for public health and community benefit.

While KT is the responsibility of both researcher and end-user, public health researchers can undertake or lead a range of actions to improve the likelihood that their research will have an impact on health policy or practice—for example, by including adequate engagement of end-users throughout the research production process, or the use of comprehensive research dissemination strategies to communicate findings to end-users. In this study we are interested in describing and examining the KT strategies undertaken by public health researchers. Specifically, the aim of this study was to describe (1) the impact of setting-based public health intervention trials; (2) the association between characteristics of trials and their impacts; and (3) the association between the use of KT strategies and trial impacts.

## Methods

### Design and sample

We conducted an international survey of authors of scientific manuscripts reporting the effects of public health interventions. We compiled a list of such researchers using author details of trials included in Cochrane systematic reviews of preventive health interventions targeting nutrition, physical activity, sexual health, tobacco use, alcohol or substance use in any organization published between 2007 and 2017. Trials that were published between 2007 and 2017 (to allow time to accrue a policy/practice impact), tested a setting-based invention (e.g. the intervention is delivered via an organization such as a hospital or school) and addressed one of the aforementioned public health targeted health risks were eligible for inclusion.

### Recruitment and data collection

We extracted author contact details, including email addresses and telephone numbers from trials included within Cochrane reviews that met the study eligibility criteria. This trial served as the reference trial (and intervention) for the study. The corresponding author listed on the included trial manuscript were invited to participate in a survey via computer-assisted telephone interview (CATI) or online via the REDCap application [21].

Up to three reminders were used to maximize response rates [22]. In instances of nonresponse, other members of the author team were approached to participate.

### Measures

The survey required authors to report KT actions undertaken and impacts specific to the reference trial. Survey items were developed based on the available literature, relevant theoretical frameworks, and feedback and pilot testing with members of the target population (i.e. researchers) [5, 6, 23–32].

*Characteristics of the trial* were extracted from the included reference trial manuscript by two members of the research team (SG and KM). Extracted data included year, study design, country, community setting, health behaviour/s targeted by the intervention and funding.

### Effectiveness of the intervention

The effect of the reference trial intervention on the primary and secondary trial outcomes was extracted from the trial manuscript or from other published reports. For trials where the primary outcome was not specified, the outcome used in the sample size calculation was used. Consistent with other research in the field, an intervention was defined as “effective” if the trial reported a significant (*p* < 0.05) effect in the hypothesized direction on the primary outcomes for the target population (not a subgroup thereof) [33, 34]. As the findings of secondary outcomes in trials are intended to be hypothesis-generating [35], trials were defined as “potentially effective” if there was no significant effect on the primary outcomes but significant effects in the desired direction on one or more secondary outcomes [35].

### Risk of bias

Risk of bias data was extracted from the Cochrane review from which the reference trial was sourced. We defined trials as having lower risk of bias if 50% or more of the risk-of-bias domains were classified as low risk of bias.

### Research impact on public health policy and practices

Survey items assessing the health policy and practice impacts of trials were developed based on the Payback framework reported by Cohen et al. [5], as well as prior impact studies [6, 23–32] and the impacts assessed in schemes including the United Kingdom’s Research Excellence Framework [36]. Specifically, these impacts included citation in policy documents or announcements, government reports, education or training materials, guidelines, professional textbooks or court rulings; formal endorsement of the intervention by government (nongovernmental) organizations; use in policy or practice decision-making; or use in the development of a commercial resource or service.

For each of the survey items, participants were asked to report “yes/no/unsure” as to whether (to the best of their knowledge) their intervention trial had achieved any of the impacts listed (Fig. 2). Participants were also asked to provide details, documentation or sources that could be used to verify their reported research impacts. This was designed to increase the accuracy of reporting as well as assist with verifying the self-reported results, methods which are consistent with previous studies on research impact [37–39]. Independent verification of the research impact reported by a random sample of 20 participants was conducted by the research team (KM). Verification was conducted using targeted web searches and cross-checking any identified verification documents with survey responses.

### KT actions and strategies domains

A list of KT actions were developed to reflect the relevant sub-domains of the KTA Framework [40]. Two sub-domains from the action cycle, “monitor knowledge use” and “evaluate outcomes”, were merged due to the similarities between these processes. The survey included 57 items assessing researcher translation actions across eight broad KT strategy domains. The list of the domains, all survey items and response options, and the frequency of responses are available in the supplementary material [see Additional file 1] and are summarized by KT strategy domain below.

Involvement of end-users (KT strategy domain 1): 13 items assessed the involvement of end-users in the trial, including who were involved and the extent to which they were engaged across each phase of the research process.

Identifying an end-user problem (KT strategy domain 2): One item assessed whether the original idea for the research was formulated based on an evaluation of a pre-existing program, as well as the expertise of the researchers, issues identified by end-users/stakeholders, or a combination of researcher interests and end-user needs.

Adapt knowledge to local context (KT strategy domain 3): Four items assessed whether the intervention was adapted, how involved end-users were in adapting the intervention and what research methods were used to make the intervention more compatible with local context.

Assess barriers to knowledge use (KT strategy domain 4): Five items assessed the extent to which individual-, organizational-, community- and political-level barriers were perceived to have affected the use of the intervention and the extent to which the intervention was modified to address these barriers.

Support to tailor and implement interventions (KT strategy domain 5): Six items assessed whether, and to what extent, staff from the target setting were trained in the intervention protocol, received feedback regarding protocol adherence, received refresher or booster training and were encouraged to contact the research team for assistance.

Evaluate outcomes and monitor knowledge use (KT strategy domain 6): Nine items assessed whether the trial evaluated changes in outcomes (effects), reach, implementation, cost, use of nonmonetary resources, adverse events, acceptability, or internal and external factors that may have impacted on the effect of the intervention.

Products and tools (KT strategy domain 7): Respondents indicated whether each of the following 11 mediums were used to disseminate their trial findings: lay summary, presentation to end-users, knowledge brokers, education workshops, education materials, media release, institutional or study website, social media, research report, academic conference or workshop.

Sustain knowledge use (KT strategy domain 8): Eight items assessed the extent to which the following were achieved during the trial: endorsement of the intervention by end-users, training of staff to deliver the intervention within their existing roles, commitment from managers, integration of the intervention into policies, use of existing resources to support delivery, adapting the intervention within funding and resources available, follow-up assessment if changes were sustained, and maintenance of partnership networks with end-users.

### Analysis

All data analysis was conducted in SAS v9.3 software. Descriptive statistics are presented as frequencies and percentages for categorical variables, and means and standard deviations (SD) for continuous variables.

To assess the policy and practice impacts of included trials, we calculated the frequency, percentage and corresponding 95% confidence interval (CI) of respondents indicating that their trial had at least one of the listed policy or practice impacts, and the same statistics were used for each of the nine individual policy and practice impacts. We also calculated the number of policy or practice impacts reported for each trial.

We created a KT strategy score for each strategy domain (except domain 2), by summing the response options within each domain (see footnote in Table 2 for scoring). To allow for comparison between domains, scores were standardized (out of 100), with higher scores representing greater KT actions within a strategy domain. A KT strategy domain score was not created for domain 2 because the response options were categorical rather than ordinal. We then calculated a total KT strategy domain score, where one point was awarded for each domain scored above the 50th percentile for that domain. For domain 2, one point was awarded if a participant indicated that the original idea for the intervention was formulated in full or in part by an end-user. This produced a total KT score ranging from 0 to 8.

The association between trial characteristics (trial quality, effectiveness, settings, targeted health risk), KT strategies and public health impact was assessed via logistic regression. Univariate and multivariable logistic regressions were conducted with the following independent variables: effectiveness of the intervention, risk of bias, intervention setting and total KT score. The measure of impact (percent of authors reporting at least one impact of their trial) was the dependent variable. The unadjusted and adjusted odds ratios (OR) and 95% CIs are reported for each characteristic, along with the *p*-value from the multivariable model. The multivariable (adjusted) model included each of the independent variables of interest. Due to the significant association found between research impact on public health policy and practice and total KT strategy domain score, exploratory univariate analyses were also conducted to assess the strength and association between each specific KT strategy domain and research impact.

## Results

**Fig. 1:**
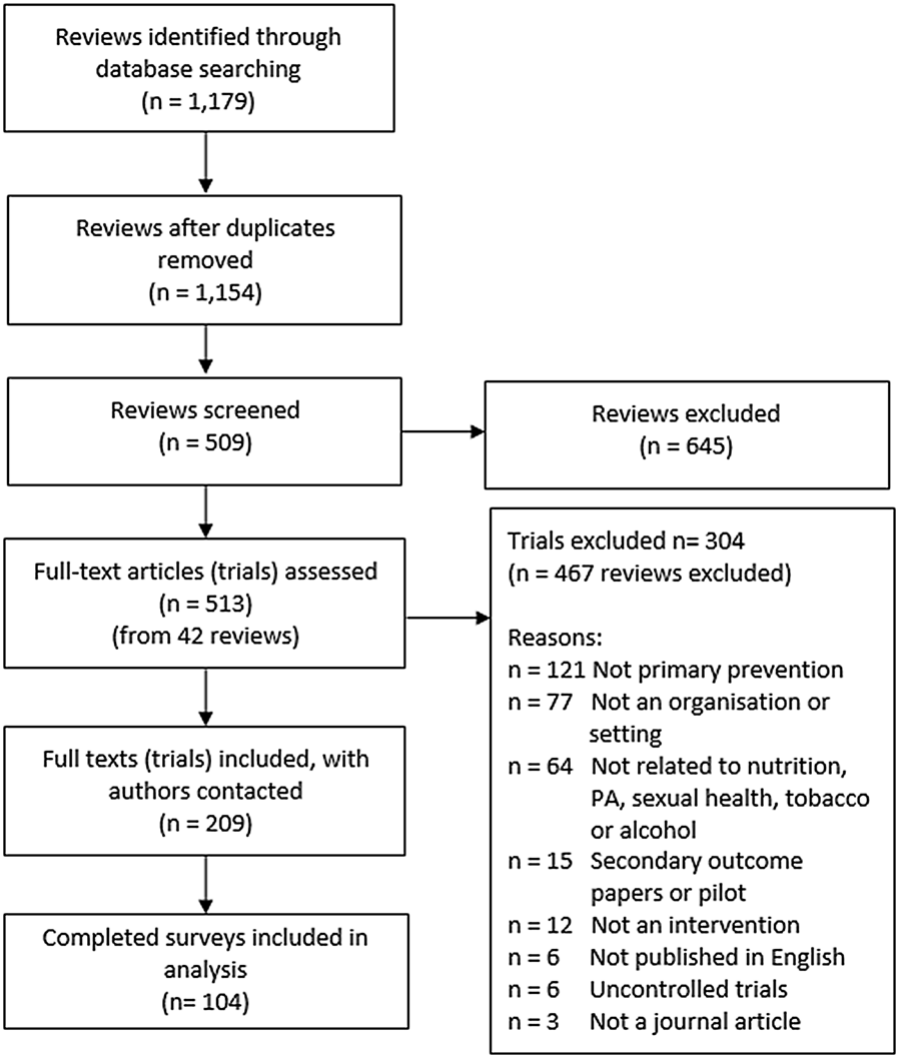
Schematic illustrates the flow, and final selection of reviews included in our study

We identified 208 trials as eligible and prepared a list of corresponding authors and coauthors (Fig. 1). Authors from 104 of these trials (50% completion rate) completed the survey. Most trials conducted by included authors were randomized controlled trials (RCTs; 83%), were conducted in North America (34%), were effective with respect to their primary outcome (68%), and assessed the effects of nutrition and/or physical activity interventions (55%) (Table 1). Most studies had a lower (36%) or unclear risk of bias (36%), while 29% had a higher risk of bias (Table 1).

**Table 1:** Characteristics of the reference trials undertaken by participating authors

Characteristics of trial	*n* = 104 (%)
Year published range 2007–2016 (mean, SD)	2010 (2.47)
Study design	
RCT	86 (83)
Other controlled trial	18 (17)
Health risk targeted	
Nutrition	13 (13)
Physical activity	22 (21)
Physical activity and nutrition	22 (21)
Sexual health	15 (14)
Smoking	12 (12)
Substance use (including substance use and another health behaviour)	20 (19)
Setting	
Community	15 (14)
Education	70 (67)
Medical	9 (9)
Worksites	7 (7)
Other	3 (3)
Country	
Europe	34 (33)
North America	35 (34)
Oceania	18 (17)
Other	17 (16)
Effective intervention	71 (68)
Potentially effective	22 (21)
Lacks evidence of effectiveness	11 (11)
Risk of bias	
Low risk of bias	37 (36)
High risk of bias	30 (29)
Unclear risk of bias	37 (36)
Funding	
Government	78 (75)
Nongovernmental	15 (14)
Not reported	11 (11)

### The proportion of setting-based public health interventions that reported a public health impact

**Fig. 2:**
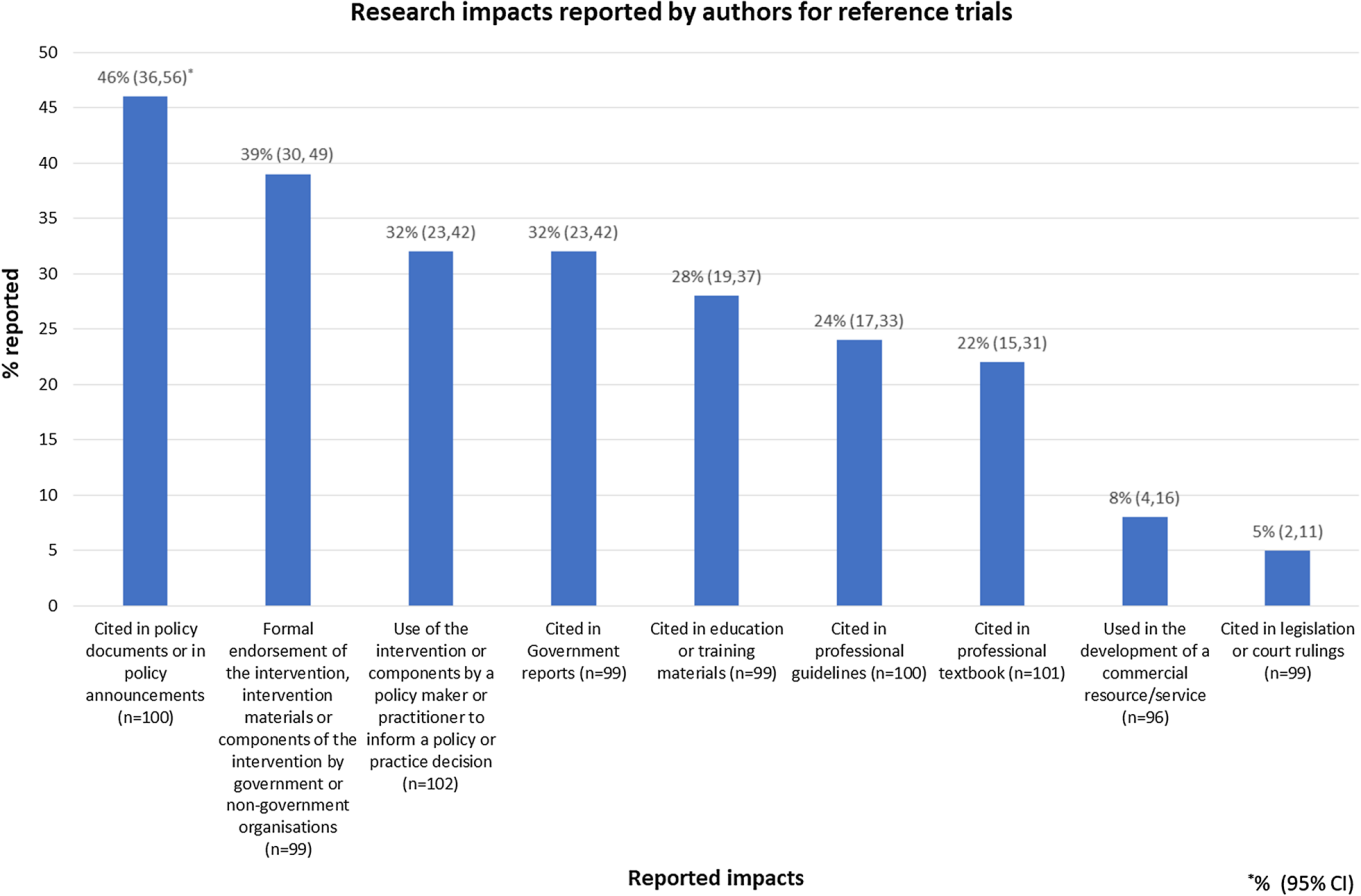
Research impact on public health policy and practice reported by authors for reference trials

In total, 65% of trials reported one or more research impacts on public health policy and practice (*n* = 66; two trials were missing a response). The median number of research impacts reported by trials was 2 (range 1–8). The most frequently reported impact was citation in a policy document or announcement (46%), while the least frequently reported impact was citation in legislation or court rulings (5%) (Fig. 2).

Independent verification of the reported research impacts on public health policy and practice was carried out for 20 randomly selected trials. Five of the reported research impacts could not be verified through documentation (e.g. endorsement). However, at least one research impact on public health policy and practice could be verified from 15 (70%) of included trials reporting one or more impact.

There was variability in the standardized KT strategy domain scores (Table 2). KT strategy domain 5 recorded the highest mean standardized domain score (mean = 71.79, SD = 27.05), indicating that triallists were particularly active in incorporating KT strategies relating to support in tailoring and implementing interventions. Conversely, the lowest standardized domain score was for involving end-users (KT strategy domain 1). The mean total domain KT strategy score was 4.18.

**Table 2:** Descriptive statistics for the standardized domain and total KT scores

KT strategy domain	No.	Mean (SD)	Median (min, max)
Involvement of end-users	102	41.68 (22.91)	41.99 (3.85, 100.0)
Adapt knowledge to the local context	100	65.58 (29.16)	75.00 (0, 100)
Assess barriers to knowledge use	99	59.77 (29.34)	62.50 (0, 100)
Support to tailor and implement interventions	95	71.79 (27.05)	75.00 (0, 100)
Evaluate outcome and monitor knowledge use	101	65.88 (19.89)	66.67 (11.11, 100)
Products and tools	100	51.84 (22.59)	54.55 (0, 100)
Sustain knowledge use	97	66.40 (24.69)	68.75 (0, 100)
Total KT score^a^	89	4.18 (2.06)	4.00 (0, 8)

^a^Total KT score ranges from 0 to 8. Response options were scored as 0 = “not at all”, 1 = “a little”/“consulted” or 2 = “substantially”/“member of the research team”, except for domain 7, where the number of dissemination techniques was summed to obtain a total score

### Association between trial characteristics, KT strategies and trial impact

There was no statistically significant association between trial characteristics and impact (Table 3). Only the total KT strategy domain score was found to be statistically significantly related to a trial reporting at least one policy or practice impact. Specifically, for every one unit increase in the total KT score, the odds of a trial reporting a policy or practice impact increased by approximately 30% (OR = 1.30, 95% CI: 1.02, 1.66; *p* = 0.031). Exploratory subgroup univariate analysis also revealed that standardized KT scores for domain 5 “support to tailor and implement interventions” (OR = 1.02, 95% CI: 1.00, 1.03; *p* = 0.047) and domain 7 “products and tools” (OR = 1.05, 95% CI: 1.02, 1.08; *p* < 0.001) were significantly associated with impact.

**Table 3:** Association between trial characteristics, KT strategies and trial impacts

Characteristic	Category level	At least one impact (*n* = 66) *n* (%) or mean (SD)	Unadjusted OR (95% CI)	Adjusted OR (95% CI)^a^	*p*-value from adjusted model
Trial effectiveness	Lacks evidence of effectiveness	6 (55%)			0.57
	Effective	43 (62%)	1.38 (0.38, 4.97)	0.99 (0.19, 5.12)	
	Potentially effective	17 (77%)	2.83 (0.60, 13.35)	1.98 (0.28, 13.93)	
Risk of bias	High risk or unclear	44 (67%)			0.64
	Low risk	22 (61%)	0.79 (0.34, 1.83)	1.31 (0.43, 4.01)	
Setting	Community and worksites	9 (41%)			0.056
	Medical and other	8 (67%)	2.89 (0.66, 12.57)	3.03 (0.49, 18.75)	
	Education	49 (72%)	3.73 (1.37, 10.14)	5.03 (1.34, 18.83)	
Health behaviour	Nutrition and physical activity	35 (61%)			0.89
	Sexual risk and substance use	31 (69%)	1.39 (0.61, 3.18)	1.07 (0.40, 2.88)	
Total KT score		4.49 (2.04)	1.25 (1.00, 1.56)	1.30 (1.02, 1.66)	0.031*
Individual domain scores					
Involvement of end-users		43.4 (22.8)	1.01 (0.99, 1.03)		
Adapt knowledge to the local context		66.6 (26.9)	1.00 (0.99, 1.02)		
Assess barriers to knowledge use		61.3 (30.0)	1.01 (0.99, 1.02)		
Support to tailor and implement interventions		76.0 (24.0)	1.02 (1.00, 1.03)*		
Evaluate outcome and monitor knowledge use		68.7 (20.1)	1.02 (1.00, 1.04)		
Products and tools		59.5 (20.3)	1.05 (1.02, 1.08)*		
Sustain knowledge use		69.8 (20.6)	1.02 (1.00, 1.03)		

*p < 0.05

^a^Model adjusted for trial effectiveness, risk of bias, setting, health behaviour and total KT score

## Discussion

### The impact of setting-based public health intervention trials

In this study, we found that almost two thirds of 104 public health research trials had one or more policy or practice impacts. Public health researchers were actively engaged in a range of research translation activities associated with their research trial, and greater KT activities were associated with greater likelihood of impact. The findings suggest that trials of public health interventions are frequently used to inform health decision-making, and that investment in such research is yielding benefits to the community.

### The association between characteristics of trials and their research impact on public health policy and practice

In comparison to previous estimates, the proportion of trials in this study demonstrating research impact on public health policy and practice (65%) was far higher than that reported by Cohen and colleagues in a 2015 study that had an impact on health policy or practice (16%) [5]. However, our estimates were more in line with those reported in 2013 by Milat and colleagues, where 59% of health promotion research funded by a government demonstration grant scheme had “moderate” to “high” policy or practice impacts [6]. These differences may be due to differences in measures of impact between these studies. Similarly, differences in the periods in which impact was assessed may also contribute to this variation. Specifically, the longer follow-up period from trial funding or completion in our study and that undertaken by Milat and colleagues provide more opportunities for impact to occur. The variability between studies may also represent the uncertainty of earlier estimates given samples of just 17–18 trials [5, 6]. Nonetheless, the findings of this study, undertaken on a relatively large scale (*n* > 100), suggest that public health trials frequently have demonstrable impact and may do so more frequently than has been reported in other health research disciplines, where estimates of impact on healthcare delivery and decision-making are 14% [8–10, 41].

### The association between the use of KT strategies and trial impacts

Our findings are consistent with previous studies reporting researcher engagement in KT activities [17, 42]. Nonetheless there remains considerable scope for improvement, particularly in actions related to the engagement of end-users, where standardized domain scores were lowest. Greater KT activity, as assessed by total KT strategy domain score, was also associated with increased odds of trial impact. Post hoc examination of KT domain scores suggests that KT actions focused on providing tailored support to facilitate program implementation and the use of research products and tools to disseminate findings may be the most influential in achieving impact. However, the reported effect sizes for these domains were small. Given the paucity of experimental studies examining the effectiveness of KT approaches for public health interventions [43], and as we are unaware of quantitative studies describing the association between specific KT activity and impact, such findings are difficult to contextualize. Nonetheless, the findings underscore the importance of KT approaches to achieving improvements in knowledge use, and highlight the need for further research to identify which KT strategies may be most beneficial across different contexts.

### Future research

Previous synthesis of case studies and mixed-methods research have reported that research quality and trials with statistically significant positive (beneficial) effects facilitate the use and impact of research, findings that were inconsistent with the results of our study [42, 44]. This could be attributed to changes in the KT behaviour of researchers, who may perceive less value in nonsignificant findings and be less engaged in promoting trial findings [42], or to the tendency for nonsignificant or less rigorous research to be published in lower-impact academic journals, which may impede their visibility to end-users [6, 45]. However, we found no evidence of association between trial quality and statistical significance with measures of impact in this study. Further research is required to explain these apparent contrasting findings.

### Strengths and limitations

This is one of few studies to quantitatively describe the impact of public health research trials and KT strategies associated with them, and the largest study to our knowledge to do so. The findings of the study, however, should be interpreted in the context of a number of limitations. First, the study sampled setting-based trials of interventions for selected health risks included in Cochrane reviews. We did not examine publication bias from the original reviews, and as such are uncertain whether this sample is biased for studies which are more likely to produce positive findings. The extent to which the findings of the study may generalize to trial designs not typically included in Cochrane reviews, unpublished trials or interventions targeting health risks ineligible for this study is unknown. Second, the response rate (50%) was low, and may increase the risk of bias due to non-participation, for example, among authors of trials that may be less likely to have had a policy or practice impact. However, researchers are typically reluctant research participants, and the response rate achieved in this study was higher than rates reported in similar surveys (typically less than 30%) [46–48]. The interventions included in this study also span a decade. For more recent trials, the KT processes may not be complete, while for others such activities and reported impacts may have occurred some time ago. Although the use of an impact verification process provides some evidence of the reliability of the reported impact, recall bias is likely, particularly among older trials [49]. As a measure of methodological robustness, we classified trials (as higher or lower risk of bias) using a numeric count of the risk-of-bias domains reported by their source Cochrane review. Studies may have been classified differently had a more sophisticated process based on consideration of individual study characteristics and bias domains been undertaken [50]. Finally, included trials may have had impacts on health policy or practice that were unknown to researchers. If this was the case, the reported level of research impact on public health policy and practice may represent an underestimate.

## Conclusions

The findings suggests that public health intervention trials frequently report policy and practice impacts and support researcher engagement in KT strategies across each phase of the research process to improve the likelihood of achieving impact. While the relative benefit of specific KT activities remains largely unknown, prospective studies with rigorous methods for capturing trial impacts would provide more rigorous evidence to guide future KT efforts.

## Supplementary Information

**Additional file 1. **Survey questions, domains and items. Details the development of survey domains, including a description of the survey items within each domain. The contents of the survey are presented, followed by a summary of the results—frequency and percentage of respondents endorsing each of the individual items from the KT domains.

## Data Availability

The datasets used and/or analysed during the current study are available from the corresponding author on reasonable request.

## Abbreviations

KT: Knowledge translation
KTA: Knowledge-to-Action Framework
CATI: Computer-assisted telephone interview
CI: Confidence interval
