# Supplementary material for: Increased use of knowledge translation strategies is associated with greater research impact on public health policy and practice: an analysis of trials of nutrition, physical activity, sexual health, tobacco, alcohol and substance use interventions
Source: Health Res Policy Syst. 2022 Jan 31;20:15. doi: 10.1186/s12961-022-00817-2 (PMC8805264; doi:10.1186/s12961-022-00817-2)
Supplement: Supplementary file 1 — Additional file 1. Survey questions, domains and items. Details the development of survey domains, including a description of the survey items within each domain. The contents of the survey are presented, followed by a summary of the results—frequency and percentage of respondents endorsing each of the individual items from the KT domains. [file 12961_2022_817_MOESM1_ESM.docx]

**Supplementary material**

**Contents**

[Survey Questions, Domains, and Items 2](#_Toc65745765)

[**Aim 1:** The proportion of setting-based public health interventions that reported a public health impact and the impacts they are having. 2](#_Toc65745766)

[**Aim 2.** The relationship between trial characteristics (trial quality, effectiveness, settings, targeted health risk), KT strategies and public health impact. 3](#_Toc65745767)

[Results: Frequency and percentage of respondents endorsing each of the individual items from the KT domains 11](#_Toc65745768)

**The development the Domains and description of corresponding items.**

| **Domain** | **Topic assessed** | **Description of items** |
| --- | --- | --- |
| **1** | **Involvement of end-users** | All participants were asked if “end-users were involved in designing, conducting and evaluating the trial?” (response options: not at all, a little, substantially). If the participant indicated ‘a little’ or ‘substantially’, they were asked to complete another eight items assessing the extent end-users were involved in certain aspects of the trial, and six items assessing the expertise of the end-users involved. These questions were related to the Action Cycle of the KTA framework, specifically the ‘identify the problem, determine knowledge gap’ and ‘select, tailor and implementation’ sub-category.^1-9^ |
| **2** | **Identify the problem** | This domain contained one item, stating “How was the original idea for the intervention formulated?”. This item was developed by the research team based on existing literature. ^1,3-5,7-9^ |
| **3** | **Adapt knowledge to the local context** | There were four items for participants to complete regarding the collection of data to assess the context in the target setting. These were based on previously used KT strategies,^2-4,7^ enquiring about how involved end-users were in making the changes made which were necessary to implement the program in the target setting, and if data collection was conducted prior to the intervention to assess the context of the target setting. |
| **4** | **Assess barriers to use** | Five items were included to assess this sub-category of the KTA cycle.^2-6,10,11^ Participants indicated ‘not at all/a little/substantially’ to how much individual level, organisational level, community level, and political level barriers affected the use of the intervention in the target setting as well as the extent modification to intervention strategies were made to address barriers. |
| **5** | **Select, tailor and implement** | This domain included 6 items with one specifically addressing the ‘select tailor and implement’ sub-category of the KTA,^1^ and five addressing the ‘sustain knowledge use’ sub category.^1-4,6,7,10,12,13^ |
| **6** | **Evaluate outcomes/monitor knowledge use** | ‘Monitor knowledge use’ and ‘evaluate outcome’ were assessed together in Domain 7 as literature indicates these two subcategories of the KTA overlap.^14^ Participants were asked to indicate ‘not at all/a little/substantially’ if they collected data on a series of nine outcomes (e.g. effect sizes, reach, acceptability) when trialling the intervention. |
| **7** | **Knowledge Creation** | This domain contained 11 items. Developed from previous literature, ^1-5,7,8,10,11,13-15^ participants were asked to indicate how they disseminated their research findings (e.g. workshops, plain language summaries, social media, etc.). |
| **8** | **Sustain knowledge use** | Eight items were included in this domain, and were based off existing literature.^1,3,4,6-8,10,12,13^ Participants were asked to indicate ‘not at all/a little/substantially’ to a series of eight question such as “endorsement of the intervention by end-users and stakeholders” to indicate the trial’s maintenance over time. |

# Survey Questions, Domains, and Items

## **Aim 1:** The proportion of setting-based public health interventions that reported a public health impact and the impacts they are having.

| **Has the intervention had any of the following impacts?** | | | |
| --- | --- | --- | --- |
| 1. Citation in policy documents or in policy announcements | - Yes | - No | - Unsure |
| 1. Citation in professional guidelines | - Yes | - No | - Unsure |
| 1. Citation in professional textbook | - Yes | - No | - Unsure |
| 1. Citation in education or training materials | - Yes | - No | - Unsure |
| 1. Citation in popular press | - Yes | - No | - Unsure |
| 1. Citation in social media (e.g. Twitter, Facebook) | - Yes | - No | - Unsure |
| 1. Citation in legislation or court rulings | - Yes | - No | - Unsure |
| 1. Citation in government reports | - Yes | - No | - Unsure |
| 1. Use of the intervention or intervention components by a policy maker or practitioner to inform a policy or practice decision | - Yes | - No | - Unsure |
| 1. Presentation of research findings to the community | - Yes | - No | - Unsure |
| 1. Used in the development of a commercial resource / service | - Yes | - No | - Unsure |
| 1. Formal endorsement of the intervention, intervention materials or components of the intervention by government or non-government organisations | - Yes | - No | - Unsure |

## **Aim 2.** The relationship between trial characteristics (trial quality, effectiveness, settings, targeted health risk), KT strategies and public health impact.

| **Domain 1 – Involvement of end-users** | | | | | |
| --- | --- | --- | --- | --- | --- |
| 1. **To what extent were end-users involved in designing, conducting and evaluating the trial?**   ***Please note that end-users who were investigators or members of the research team should still be considered when answering this question.***  **Just a reminder end-users include:** Person(s) or organisation(s) that are expected to directly use or benefit from the intervention. This could include: students, clients, patients, staff members, health care providers, policy makers. | - Not at all (Go to Q28) | | - A little | | - Substantially |
| *Participants who answer a little or extensively to the previous question will be asked about the following specific strategies.* | | | | | |
| **To what extent were end-users involved in the following aspects of the trial?** | | | | | |
| Formulation of the research question | - Not at all | - A little | | - Substantially | |
| Determining the study methodology | - Not at all | - A little | | - Substantially | |
| Developing the intervention | - Not at all | - A little | | - Substantially | |
| Delivering the intervention | - Not at all | - A little | | - Substantially | |
| Data collection | - Not at all | - A little | | - Substantially | |
| Data analysis | - Not at all | - A little | | - Substantially | |
| Interpretation of trial findings | - Not at all | - A little | | - Substantially | |
| Dissemination of trial findings | - Not at all | - A little | | - Substantially | |

| **SECTION 2 – Design, development and evaluation of the intervention trial** | | | |
| --- | --- | --- | --- |
| **Domain 1 – Involvement of end-users** | | | |
| 1. **To what extent were individuals with the following expertise involved in the design, conduct and evaluation of the trial?**   Please note members of the research team are defined as someone who made a substantive contribution to the development, conduct and/or analysis of the trial. Someone who was narrowly involved in one of these aspects of the trial, and who offered only advice or expertise on a very specific issue should be considered ‘consulted’.  (*Please select all that apply*) | | | |
| Researchers with expertise relating to the content area of the trial (e.g. nutrition, physical activity, smoking) | - Not at all | - Consulted | - Member of the research team |
| Staff members from the target setting  (e.g. teachers, health care providers) | - Not at all | - Consulted | - Member of the research team |
| Members of the target population  (e.g. students, clients, patients) | - Not at all | - Consulted | - Member of the research team |
| Representatives from the types of organisations where the intervention was trialled (e.g. school principals, workplace managers, hospital administrators) | - Not at all | - Consulted | - Member of the research team |
| Representatives from the types of organisations responsible for directing, supporting or financing the organisations where the intervention was trialled (e.g. department of education, local health service) | - Not at all | - Consulted | - Member of the research team |
| Policy makers | - Not at all | - Consulted | - Member of the research team |

| **SECTION 2 – Design, development and evaluation of the intervention trial** | |
| --- | --- |
| **Domain 2 – Identify the problem** | |
| 1. **How was the original idea for the intervention formulated? (*Please select the most appropriate option*)** | |
| It was a pre-existing program or service that required evaluation |  |
| It was predominately based on the interest of the researcher/s |  |
| In direct response to an issue identified by end-users and/or stakeholders |  |
| A combination of researcher interests and end-user and/or stakeholder needs |  |

| **SECTION 2 – Design, development and evaluation of the intervention trial** | | | | | |
| --- | --- | --- | --- | --- | --- |
| **Domain 3 – Adapt knowledge to the local context** | | | | | |
| 1. **How involved were end-users in adapting the intervention strategies to make them more compatible with the target setting?** | | - Not at all | - A little | - Substantially | |
| 1. **How involved were end users in adapting the research methods to make them more compatible with the target setting?** | | - Not at all | - A little | - Substantially | |
| 1. **Prior to conducting the trial did you collect data to assess the context of the target setting? By context we mean usual operational routines and environment, structures of behaviours within the local setting that may be relevant for the intervention.** | | - Not at all   (Go to Q35) | - A little | - Substantially | |
| *Participants who answer a little or substantially to the previous question will be asked the following question Q35.* | | | | |  |
| 1. **Did you use this data to inform the development of the intervention strategies?** | - Not at all (Go to Q35) | | - A little | - Substantially |  |
| *Participants who answer a little or substantially to the previous question will be asked about the following specific strategies.* | | | | |  |

| **SECTION 2 – Design, development and evaluation of the intervention trial** | | | |
| --- | --- | --- | --- |
| **Domain 4 – Assess barriers to knowledge use** | | | |
| 1. **When designing the intervention …** **did you assess any of the following types of barriers that may affect the use of the intervention in the target setting?** | | | |
| Individual level  (e.g. provider skills, knowledge, attitude, opinions, characteristics of target population) | - Not at all | - A little | - Substantially |
| Organisational level  (e.g. infrastructure resources, financial resources, management structure, organisational priorities) | - Not at all | - A little | - Substantially |
| Community level  (e.g. community views and preferences, local support, views of opinion leaders) | - Not at all | - A little | - Substantially |
| Political level  (e.g. policies, legislation, governance) | - Not at all | - A little | - Substantially |
| 1. **To what extent did you make modifications to your broad intervention strategy or approach in order to address any identified barriers?** | - Not at all | - A little | - Substantially |

| **SECTION 2 – Design, development and evaluation of the intervention trial** | | | |
| --- | --- | --- | --- |
| **Domain 5 – Select, tailor and implement** | | | |
| 1. **Before beginning the trial did you communicate the potential benefits of the intervention to end-users?**   **Benefits may include:** reduced cost, health benefits for the target population or wider community, increased organisational efficacy. | - Not at all | - A little | - Substantially |
| 1. **Were staff from the target setting involved in the delivery of the intervention?** | - Not at all (Go to Q39) | - A little | - Substantially |
| *Participants who answer a little or extensively to the previous question will be asked about the following specific strategies.* | | | |
| **To what extent were staff from the target setting:** | | | |
| Trained in the protocol | - Not at all | - A little | - Substantially |
| Provided with ongoing feedback about adherence to the protocol | - Not at all | - A little | - Substantially |
| Provided with refresher or booster training | - Not at all | - A little | - Substantially |
| Encouraged to contact a research team member for help when needed | - Not at all | - A little | - Substantially |

| **SECTION 2 – Design, development and evaluation of the intervention trial** | | | |
| --- | --- | --- | --- |
| **Domain 6 – Evaluate outcomes / Monitor Knowledge Use** | | | |
| 1. **During the trialling of the intervention did you collect data on the following?** | | | |
| Changes in outcomes and effect sizes | - Not at all | - A little | - Substantially |
| Reach of the intervention to the target population | - Not at all | - A little | - Substantially |
| Intervention implementation (e.g. adherence to study protocol, fidelity of delivery) | - Not at all | - A little | - Substantially |
| The monetary cost of the intervention to staff, end-users and the overall organisation | - Not at all | - A little | - Substantially |
| The non-monetary resource implications of the intervention to staff, end-users and the overall organisation | - Not at all | - A little | - Substantially |
| Unforeseen incidents or adverse events recorded | - Not at all | - A little | - Substantially |
| Acceptability of the trial to staff, end-users and the overall organisation | - Not at all | - A little | - Substantially |
| Internal factors that may affect the impact of the intervention on the main outcomes (e.g. organisational structure and policies, staff behaviour and beliefs) | - Not at all | - A little | - Substantially |
| External factors that may affect the impact of the intervention on the main outcomes (e.g. change in laws or governmental policies) | - Not at all | - A little | - Substantially |

| **SECTION 2 – Design, development and evaluation of the intervention trial** | |
| --- | --- |
| **Domain 7. Products and tools** | |
| 1. **How did you disseminate the findings from your trial?**   *(please select all that apply)* | |
| Plain language or lay summary |  |
| Targeted presentations to end-users |  |
| Knowledge broker used to communicate findings to end-users |  |
| Education workshops conducted with end-users |  |
| Education materials on how to use the study findings |  |
| Media releases |  |
| Results posted on institutional or study website |  |
| Results posted on social media platforms (e.g. Facebook, Twitter, blogs) |  |
| Publication of results in peer reviewed journals |  |
| Research reports |  |
| Presented at academic conferences, workshops or forums |  |

| **SECTION 2 – Design, development and evaluation of the intervention trial** | | | |
| --- | --- | --- | --- |
| **Domain 8 – Sustain knowledge use** | | | |
| 1. **To what extent were the following achieved during the trial?** | | | |
| Endorsement of the intervention by end-users and stakeholders | - Not at all | - A little | - Substantially |
| Staff within the target setting were trained to deliver the intervention within their existing roles | - Not at all | - A little | - Substantially |
| Commitment from managers to support the intervention within their existing roles | - Not at all | - A little | - Substantially |
| Integration of the intervention within existing policies within the target setting | - Not at all | - A little | - Substantially |
| Use of existing resources within the target setting to support the delivery of the intervention | - Not at all | - A little | - Substantially |
| Adapting the trial to ensure that it could be implemented within the funding and resources available within the organisation | - Not at all | - A little | - Substantially |
| Follow-up assessments (once the intervention period had ended) to measure if changes were sustained over time | - Not at all | - A little | - Substantially |
| Maintenance of partnership networks with end-users | - Not at all | - A little | - Substantially |

# Results

# Frequency and percentage of respondents endorsing each of the individual items from the KT domains

| **Question** | **Response items** | **Response option** | **n (%)** |
| --- | --- | --- | --- |
| **Domain 1: Involvement of end-users** | | | |
| **To what extent were end-users involved in the following aspects of the trial?** | Formulation of the research question (n=100) | Substantially | 24 (24%) |
|  | Determining the study methodology (n=101) | Substantially | 20 (20%) |
|  | Developing the intervention (n=101) | Substantially | 47 (47%) |
|  | Delivering the intervention (n=100) | Substantially | 61 (61%) |
|  | Data collection (n=102) | Substantially | 26 (25%) |
|  | Data analysis (n=102) | Substantially | 6 (6%) |
|  | Interpretation of trial findings (n=100) | Substantially | 10 (10%) |
|  | Dissemination of trial findings (n=101) | Substantially | 25 (25%) |
| **To what extent were individuals with the following expertise involved in the design, conduct and evaluation of the trial?** | Staff members from the target setting (e.g. teachers, health care providers) (n=102) | Member of the research team | 30 (29%) |
|  | Members of the target population (e.g. students, clients, patients) (n=100) | Member of the research team | 6 (6%) |
|  | Representatives from the types of organisations where the intervention was trialled (e.g. school principals, workplace managers, hospital administrators) (n=99) | Member of the research team | 11 (11%) |
|  | Representatives from the types of organisations responsible for directing, supporting or financing the organisations where the intervention was trialled (e.g. department of education, local health service) (n=97) | Member of the research team | 13 (13%) |
|  | Policy makers (n=100) | Member of the research team | 8 (8%) |
| **Domain 2: Identify the problem** | | | |
| **How was the original idea for the intervention formulated? Please select the most appropriate option (n=102)** | It was a pre-existing program or service that required evaluation |  | 14 (14%) |
|  | It was predominately based on the expertise of the researcher/s |  | 27 (26%) |
|  | In direct response to an issue identified by end-users and/or stakeholders |  | 4 (4%) |
|  | A combination of researcher interests and end-user and/or stakeholder needs |  | 53 (52%) |
|  | Other, please specify |  | 4 (4%) |
| **Domain 3: Adapt knowledge to the local context** | | | |
| **How involved were end-users in adapting the intervention strategies to make them more compatible with the target setting? (n=99)** |  | Substantially | 49 (49%) |
| **How involved were end users in adapting the research methods to make them more compatible with the target setting? (n=100)** |  | Substantially | 30 (30%) |
| **Prior to conducting the trial did you collect data to assess the context of the target setting? By context we mean usual operational routines and environment, structures or behaviours within the local setting that may be relevant for the intervention. (n=101)** |  | Substantially | 57 (56%) |
| **Did you use this data to inform the development of the intervention strategies? (n=98)** |  | Substantially | 63 (64%) |
| **Domain 4: Assess barriers to knowledge use** | | | |
| **When designing the intervention did you assess any of the following types of barriers that may affect the use of the intervention in the target setting?** | Individual level (e.g. provider skills, knowledge, attitude, opinions, characteristics of target population) (n=98) | Substantially | 59 (60%) |
|  | Organisational level (e.g. infrastructure resources, financial resources, management structure, organisational priorities) (n=97) | Substantially | 55 (57%) |
|  | Community level (e.g. community views and preferences, local support, views of opinion leaders) (n=96) | Substantially | 36 (37%) |
|  | Political level (e.g. policies, legislation, governance) (n=96) | Substantially | 24 (25%) |
| **To what extent did you make modifications to your broad intervention strategy or approach in order to address any identified barriers? (n=94)** |  | Substantially | 38 (40%) |
| **Domain 5: Select, tailor and implement** | | | |
| **Were staff from the target setting involved in the delivery of the intervention? (n=100)** |  | Substantially | 78 (78%) |
| **Before beginning the trial did you communicate the potential benefits of the intervention to end-users? (Benefits may include: reduced cost, health benefits) (n=97)** |  | Substantially | 66 (68%) |
| **To what extent were staff from the target setting:** | Trained in the protocol (n=98) | Substantially | 62 (63%) |
|  | Provided with ongoing feedback about adherence to the protocol (n=94) | Substantially | 46 (49%) |
|  | Provided with refresher or booster training (n=92) | Substantially | 28 (30%) |
|  | Encouraged to contact a research team member for help when needed (n=97) | Substantially | 69 (71%) |
| **Domain 6: Evaluate outcomes and monitor knowledge use** | | | |
| **During the trialling of the intervention did you collect data on the following?** | Changes in outcomes and effect sizes (n=100) | Substantially | 87 (87%) |
|  | Reach of the intervention to the target population (n=96) | Substantially | 72 (75%) |
|  | Intervention implementation (e.g. adherence to study protocol, fidelity of delivery) (n=99) | Substantially | 74 (75%) |
|  | The monetary cost of the intervention to staff, end-users and the overall organisation (n=98) | Substantially | 29 (30%) |
|  | The non-monetary resource implications of the intervention to staff, end-users and the overall organisation (n=96) | Substantially | 27 (28%) |
|  | Unforeseen incidents or adverse events recorded (n=91) | Substantially | 40 (44%) |
|  | Acceptability of the trial to staff, end-users and the overall organisation (n=98) | Substantially | 70 (71%) |
|  | Internal factors that may affect the impact of the intervention on the main outcomes (e.g. organisational structure and policies, staff behaviour and beliefs) (n=98) | Substantially | 41 (42%) |
|  | External factors that may affect the impact of the intervention on the main outcomes (e.g. change in laws or governmental policies) (n=97) | Substantially | 22 (23%) |
| **Domain 7: Products and tools** | | | |
| **How did you disseminate the findings from your trial? (n=104)** | Plain language or lay summary | Selected | 67 (64%) |
|  | Targeted presentations to end-users | Selected | 71 (68%) |
|  | Knowledge broker used to communicate findings to end-users | Selected | 17 (16%) |
|  | Education workshops conducted with end-users | Selected | 41 (39%) |
|  | Education materials on how to use the study findings | Selected | 30 (29%) |
|  | Media releases | Selected | 61 (59%) |
|  | Results posted on institutional or study website | Selected | 52 (50%) |
|  | Results posted on social media platforms (e.g. Facebook, Twitter, blogs) | Selected | 7 (7%) |
|  | Publication of results in peer reviewed journals | Selected | 99 (95%) |
|  | Research reports | Selected | 53 (51%) |
|  | Presented at academic conferences, workshops or forums | Selected | 95 (91%) |
| **Domain 8: Sustain knowledge use** | | | |
| **To what extent were the following achieved during the trial?** | Endorsement of the intervention by end-users and stakeholders (n=95) | Substantially | 51 (54%) |
|  | Staff within the target setting were trained to deliver the intervention within their existing roles (n=92) | Substantially | 64 (70%) |
|  | Commitment from managers to support the intervention within their existing roles (n=95) | Substantially | 47 (49%) |
|  | Integration of the intervention within existing policies within the target setting (n=96) | Substantially | 42 (44%) |
|  | Use of existing resources within the target setting to support the delivery of the intervention (n=95) | Substantially | 54 (57%) |
|  | Adapting the trial to ensure that it could be implemented within the funding and resources available within the organisation (n=92) | Substantially | 38 (41%) |
|  | Follow-up assessments (once the intervention period had ended) to measure if changes were sustained over time. (n=98) | Substantially | 48 (49%) |
|  | Maintenance of partnership networks with end-users (n=93) | Substantially | 41 (44%) |

**References**

1. Milat AJ, Laws R, King L, et al. Policy and practice impacts of applied research: a case study analysis of the New South Wales Health Promotion Demonstration Research Grants Scheme 2000–2006. *Health Research Policy and Systems.* 2013;11(1):5.

2. Sumner A, Crichton J, Theobald S, Zulu E, Parkhurst J. What shapes research impact on policy? Understanding research uptake in sexual and reproductive health policy processes in resource poor contexts. *Health Research Policy and Systems.* 2011;9(1):S3.

3. Grimshaw JM, Eccles MP, Lavis JN, Hill SJ, Squires JE. Knowledge translation of research findings. *Implementation Science.* 2012;7(1):50.

4. Reed RL, McIntyre E, Jackson-Bowers E, Kalucy L. Pathways to research impact in primary healthcare: What do Australian primary healthcare researchers believe works best to facilitate the use of their research findings? *Health Research Policy and Systems.* 2017;15(1):17.

5. Yazdizadeh B, Majdzadeh R, Janani L, et al. An assessment of health research impact in Iran. *Health Research Policy and Systems.* 2016;14(1):56.

6. Zardo P, Collie A. Type, frequency and purpose of information used to inform public health policy and program decision-making. *BMC Public Health.* 2015;15(1):381.

7. Greenhalgh T, Fahy N. Research impact in the community-based health sciences: an analysis of 162 case studies from the 2014 UK Research Excellence Framework. *BMC Medicine.* 2015;13(1):232.

8. Kothari A, Peter N, Donskov M, Luciani T. Research impact of systems-level long-term care research: a multiple case study. *Health Research Policy and Systems.* 2017;15(1):23.

9. Tsey K, Lawson K, Kinchin I, et al. Evaluating research impact: the development of a research for impact tool. *Frontiers in Public Health.* 2016;4:160.

10. Oliver K, Innvar S, Lorenc T, Woodman J, Thomas J. A systematic review of barriers to and facilitators of the use of evidence by policymakers. *BMC Health Services Research.* 2014;14(1):2.

11. Newson R, King L, Rychetnik L, et al. A mixed methods study of the factors that influence whether intervention research has policy and practice impacts: perceptions of Australian researchers. *BMJ Open.* 2015;5(7):e008153.

12. Hanney SR, Castle-Clarke S, Grant J, et al. How long does biomedical research take? Studying the time taken between biomedical and health research and its translation into products, policy, and practice. *Health Research Policy and Systems.* 2015;13(1):1.

13. Menon A, Korner-Bitensky N, Kastner M, McKibbon K, Straus S. Strategies for rehabilitation professionals to move evidence-based knowledge into practice: a systematic review. *Journal of Rehabilitation Medicine.* 2009;41(13):1024-1032.

14. Cohen G, Schroeder J, Newson R, et al. Does health intervention research have real world policy and practice impacts: testing a new impact assessment tool. *Health Research Policy and Systems.* 2015;13(1):3.

15. Ovseiko PV, Oancea A, Buchan AM. Assessing research impact in academic clinical medicine: a study using Research Excellence Framework pilot impact indicators. *BMC Health Services Research.* 2012;12(1):478.
